# White matter damage due to pulsatile versus steady blood pressure differs by vascular territory: A cross-sectional analysis of the UK Biobank cohort study

**DOI:** 10.1177/0271678X211058803

**Published:** 2021-11-14

**Authors:** Karolina A Wartolowska, Alastair JS Webb

**Affiliations:** Wolfson Centre for Prevention of Stroke and Dementia, Nuffield Department of Clinical Neurosciences, University of Oxford, UK

**Keywords:** Small vessel disease, mean arterial blood pressure, pulse pressure, hypertension, white matter hyperintensities, diffusion tensor imaging (DTI), neurite orientation dispersion and density imaging (NODDI)

## Abstract

Small vessel disease is associated with age, mean blood pressure (MAP) and blood pressure pulsatility (PP). We used data from the UK Biobank cohort study to determine the relative importance of MAP versus PP driving white matter injury within individual white matter tracts, particularly in the anterior and posterior vascular territory. The associations between blood pressure and diffusion indices in 27 major tracts were analysed using unadjusted and fully-adjusted general linear models and mixed-effect linear models. Blood pressure and neuroimaging data were available for 37,041 participants (mean age 64+/−7.5 years, 53% female). In unadjusted analyses, MAP and PP were similarly associated with diffusion indices in the anterior circulation. In the posterior circulation, the associations were weaker, particularly for MAP. In fully-adjusted analyses, MAP remained associated with all diffusion indices in the anterior circulation, independently of age. In the posterior circulation, the effect of MAP became protective. PP remained associated with greater mean diffusivity and extracellular free water diffusion in the anterior circulation and all diffusion indices in the posterior circulation. There was a significant interaction between PP and age. This implies discordant mechanisms for chronic white matter injury in different brain regions and potentially in the associated stroke risks.

## Introduction

Small vessel disease (SVD) is associated with an increased risk of stroke,^1^,^2^ dementia,^1–3^ late-life disability,^4^ and all-cause mortality.^2^ Progression of SVD^2^ and its neuroimaging markers is strongly associated with age and hypertension.^5–9^ In middle-aged people, these changes are strongly associated with an increase in both systolic and diastolic blood pressure represented by mean arterial blood pressure and reflecting the steady-state component of the blood flow; however, later in life the effect depends on the difference between the systolic and diastolic blood pressure corresponding to the pulsatility of the blood flow.^10^ However, the effect of blood pressure on white matter integrity may vary across the brain regions due to differences in the location of white matter tracts relative to vascular supply and watershed regions. In particular, the importance of differences in blood pressure and its pulsatility between the anterior and posterior cerebrovascular circulation due to the damping effect of the blood pressure at the level of the carotid siphon^11^ is uncertain. Differences in haemodynamic mechanisms of injury between the anterior and posterior circulation may require adaptation of treatment to prevent future stroke and progression of white matter injury depending on the location of small vessel disease in individual patients.

Macrostructural white matter injury manifests as lacunar strokes, white matter hyperintensities (WMH), microbleeds, and enlarged perivascular spaces.^2^ However, early microstructural white matter damage that is evident on diffusion-weighted magnetic resonance imaging (dMRI) as altered diffusion of water molecules within and around neurites. It may be characterised as an increase in mean diffusivity (MD) and reduction in anisotropy^9,12,13^ reflecting the loss of structural integrity of white matter tracts.^14^ Diffusion changes precede the development of macrostructural changes in SVD and may provide a sensitive tool to investigate disease mechanisms in its early stages,^9^ for example, the relative contribution of different haemodynamic measures.

Therefore, in the UK Biobank cohort, we determined whether associations of mean arterial blood pressure or pulse pressure with individual white matter tracts differed and whether these associations varied between tracts located predominantly within the anterior versus posterior cerebrovascular circulation.

## Materials and methods

### Participants and data

UK Biobank is a large, prospective cohort study including demographic, lifestyle, clinical, and imaging data of 502,540 middle-aged community-based people recruited from 22 centres across the UK. Informed consent was obtained from all UK Biobank participants. The details regarding the ethical approval and governance are described on the UK Biobank website (http://www.ukbiobank.ac.uk/ethics) and the IRB approval was obtained from the North West Multi-centre Research Ethics Committee.

This study included UK Biobank participants with structural neuroimaging data, both white matter hyperintensity (WMH) volume and diffusion-weighted imaging (dMRI), and systolic and diastolic blood pressure measurements (SBP and DBP). People with a condition potentially associated with cerebral white matter damage, such as multiple sclerosis or other demyelinating disorders, cerebral infarction, encephalitis or brain abscess, brain tumour or systemic lupus erythematosus, were excluded on the basis of the codes from the 10th revision of the International Statistical Classification of Diseases and Related Health Problems (ICD-10).

UK Biobank brain MRI data were acquired on a single Siemens Skyra 3 T scanner, and the sequence parameters are available at https://biobank.ctsu.ox.ac.uk/ crystal/crystal/docs/brain_mri.pdf. T1-weighed 3D MPRAGE, FLAIR, and dMRI data were pre-processed and analysed by the UK Biobank brain imaging team using FMRIB Software Library (FSL) tools (http://www.fmrib.ox.ac.uk/fsl).

Analysed dMRI variables included diffusion tensor imaging (DTI) measures in individual white matter tracts, such as fractional anisotropy (FA) and mean diffusivity (MD) as well as neurite orientation dispersion and density imaging (NODDI) measures such as Intracellular Volume Fraction (ICVF), the Isotropic Compartment Volume Fraction (ISOVF), and the Orientation Dispersion (OD).^15^,^16^ DTI indices are sensitive to myelination and structural integrity of white matter but cannot distinguish between changes in the neurite density and changes in neurite arrangement.^12^ NODDI overcomes these limitations and provides estimates of neurite density as the ICVF and the variability of neurite orientation as OD as well as estimates of diffusion in the extracellular free water as ISOVF.^15^,^16^ In the UK Biobank, DTI data were analysed using FSL tools^17^ and the NODDI indices were derived by AMICO (Accelerated Microstructure Imaging via Convex Optimization).^16^ The white matter tracts were defined using the Juelich (JHU ICBM-DTI-81) atlas and presented for reference in Supplementary Figure 1. The tract-average DTI and NODDI measures were derived for 27 major white matter tracts.

The main explanatory variables were the mean arterial pressure (MAP) and pulse pressure (PP) calculated from the automated systolic (SBP) and diastolic (DBP) blood pressure measures. In the UK Biobank, blood pressure was measured twice by trained nurses after the participant had been at rest for at least 5 minutes in the seated position with a digital sphygmomanometer (Omron 705 IT; OMRON Healthcare Europe B.V., Hoofddorp, Netherlands) with a suitably sized cuff. When automated blood pressure values were not available, manual blood pressure was used, and when it also was missing, values from Pulsewave Analysis (PWA) were used. Measures of SBP and DBP were averaged within a visit.

As diffusion measures are affected by white matter lesions, the volume of white matter hyperintensities (WMH) was used as a covariate and used to stratify the analyses. In the UK Biobank, the volume of WMH was calculated from the T1-weighted and T2-FLAIR images using BIANCA,^18^ and the volumes of segmented white matter (WM) was estimated using FAST;^19^ both algorithms are a part of FSL.^17^ The WMH volume was divided by the total volume of WM and logit-transformed to normalise and stabilise the variance; this measure was further referred to as WMH load.

### Statistical analysis

To compare the explanatory variables with different units of measurement, continuous variables were centred and scaled before being entered into regression analyses. The effect of MAP and PP on DTI and NODDI dMRI measures within individual white matter tracts was analysed with sequential addition of covariates to assess the impact of adjustment, first, using unadjusted models (dMRI ∼ PP or dMRI ∼ MAP), then, after adjusting for age and sex (dMRI ∼ PP + age + sex OR dMRI ∼ MAP + age + sex), and, finally, after adjusting for the other blood pressure measures and cardiovascular risk factors (dMRI ∼ MAP + PP + age + sex + smoking + diabetes + antihypertensives + BPmeasure + centre). The same covariates were included in all the multivariable models. The effects were also investigated using mixed-effects models, where we had to allow for random effects due to multiple regions being included from the same individual (dMRI ∼ circulation + age + sex + MAP + PP + antihypertensives + smoking + diabetes + BPmeasure + centre + antihypertensives: MAP + antihypertensives:PP + age:MAP + age:PP + circulation:PP + circulation:MAP + (1|eid) + (1|roi)).

The effect of MAP and PP on DTI and NODDI dMRI measures within individual WM tracts was analysed, first, using unadjusted models, then, after adjusting for age and sex, and, finally, after adjusting for the other blood pressure measure, age, sex, WMH load, smoking status, diagnosis of diabetes, the source of blood pressure measurement, and an assessment centre. All modelling was performed using the concurrent explanatory variables, i.e. acquired at the same time as the MRI. Standardised coefficients between the dMRI measures and blood pressure were presented in tables, forest plots, and brain maps. The tables and plots summarised both, left and right hemisphere tracts together whereas the maps presented standardised coefficients for MAP and PP within each white matter tract separately. On the brain maps and forest plots, the strength of associations was represented on a saturation scale with red corresponding to positive associations, and blue to negative associations; for the interactions that did not reach significance the coefficients were set to zero and rendered as white. In the forest plots, the WM tracts were presented in the superior/anterior to inferior/posterior order.

The analyses were also stratified by age decade (<60, 60–70, ≥70) and tertiles of WMH load. In order to investigate the interactions between age and blood pressure and the effect of vascular territory on dMRI, a mixed-effect analysis was performed using participants and tracts as fixed factors, and adding a binary variable encoding whether a tract was within the anterior or posterior circulation; the analysis was stratified by tertiles of WMH load. The tracts supplied predominantly by the posterior circulation included: splenium of the corpus callosum, the hippocampal part of the cingulum, cerebral peduncle, corticospinal tract, pontine crossing tract, medial lemniscus, inferior, middle, and superior cerebellar peduncle. Analysed tracts and used abbreviations are presented in Supplementary Figure 1 and the list of abbreviations.

Modelling assumptions were checked using diagnostic plots of residuals. Fractional polynomials terms were used to determine the presence of any statistically significant non-linear relationships, with Bayes information criterion (BIC) used to determine whether model fit had been improved or worsened through the addition or removal of any terms. Data management and analyses were performed using R version 4.0.2 using the *data.table* package version 1.13.6, the *magrittr* package version 2.0.1, the *lme4* package version 1.1.26, and the mfp package version 1.5.2. Figures were plotted using *ggplot2* version 3.3.3 and annotated using *captioner* version 2.2.3. Additionally, *stringr* version 1.4.0 and *fst* 0.9.4 packages were used for data management. The manuscript was typeset using *knitr* version 1.30 in RMarkdown.

### Data availability

All data is available upon application to the UK Biobank by eligible researchers.

## Results

A total of 38,347 UK Biobank participants had brain magnetic resonance imaging (MRI) data available. After the exclusion of 492 due to diagnoses that could confound white matter assessment, and 822 due to missing blood pressure data, 37,041 were eligible for inclusion in analyses (Table 1).

**Table 1.** Baseline characteristics of participants.

Variable	Values at the time of MRI
N	37,041
Age, years	64.12 (7.52)
Female sex, N (%)	19,635 (53.0)
Height, cm, mean (SD)	169.07 (9.24)
Weight, kg, mean (SD)	75.94 (15.06)
BMI, kg/m^2^, mean (SD)	26.48 (4.37)
Diabetes, N (%)	1,855 (5.0)
Ex-smoker, N (%)	12,334 (33.5)
Active smoker, N (%)	1,256 (3.4)
MAP, mmHg, mean (SD)	97.86 (11.89)
PP, mmHg, mean (SD)	60.59 (15.16)
SBP, mmHg, mean (SD)	138.26 (18.72)
DBP, mmHg, mean (SD)	77.66 (10.65)
ASI, mean (SD)	9.61 (2.91)
Myocardial infarct, N (%)	169 (0.5)
Angina, N (%)	202 (0.5)
Hypertension diagnosis, N (%)	2,422 (6.5)
Antihypertensive medication, N (%)	8,269 (22.3)

Values are reported as means (standard deviation) or as numbers and frequency in %.

### Interactions between diffusion markers and the steady component of blood pressure

In unadjusted analyses, the steady component of blood pressure represented by MAP was associated with dMRI changes indicative of white matter injury, namely lower FA, higher MD, lower intracellular (ICVF), higher extracellular (ISOVF) diffusion fraction, and higher directionality of neurites (OD) (Supplementary Figure 2). The relationships were similar for FA, MD, ICVF, and ISOVF, but there was some heterogeneity in associations with neurite orientation (OD). The strength of associations was stronger in the anterior circulation, with a reversal of the direction of the effect for ICVF in the brainstem regions. After adjusting for age and sex, the effect size of MAP decreased, but the difference between the superior/anterior and inferior/posterior regions remained, especially for MD, ICVF, and ISOVF (Supplementary Figure 3).

**Figure 1.**
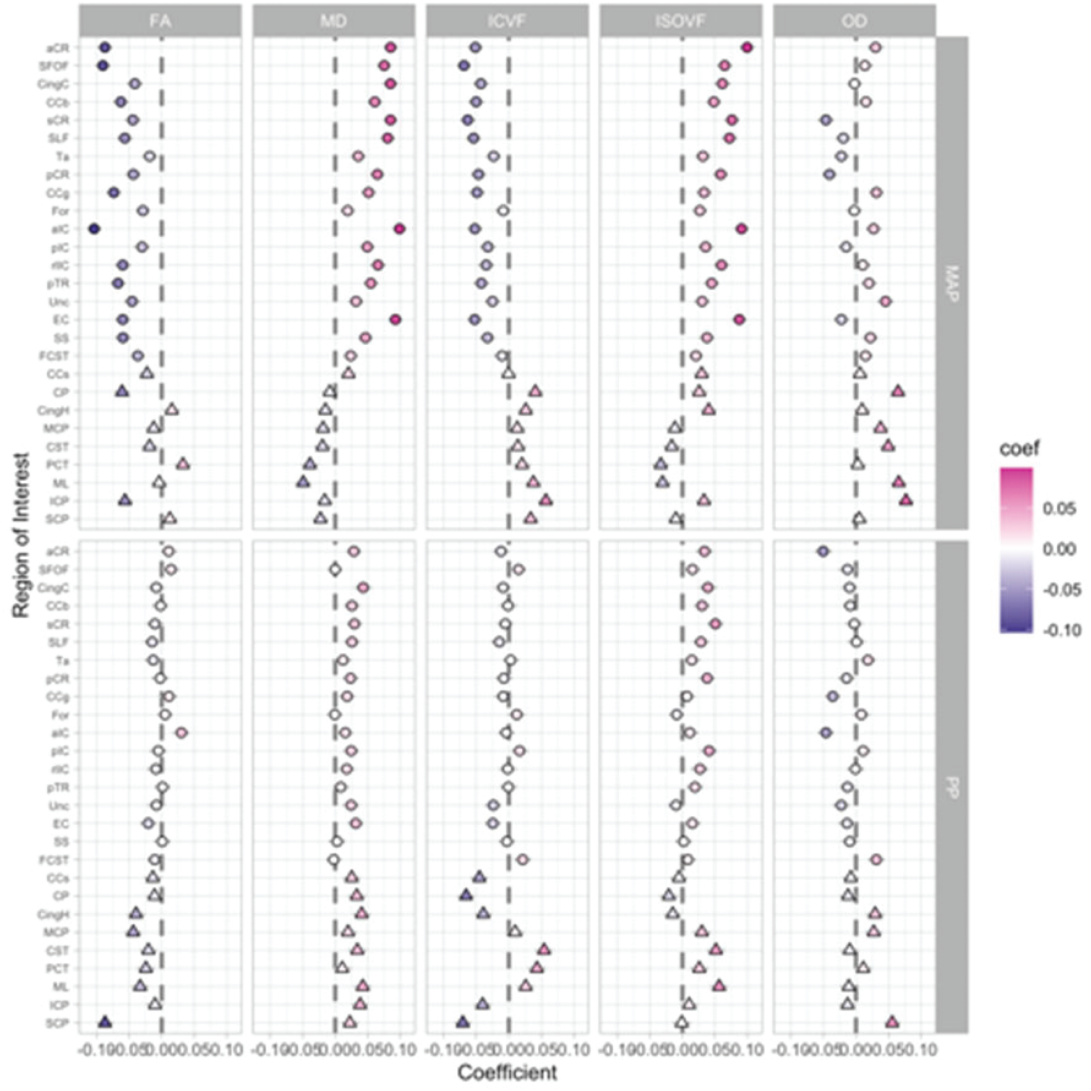
Standardised coefficients for concurrent mean arterial pressure (MAP) and pulse pressure (PP) in fully adjusted linear models with dMRI markers as outcome variables. The coefficient values are on the x axis and presented on the colour scale, with the most negative values plotted in blue and most positive in red. The individual white matter tracts are on the y axis arranged approximately in superior/anterior to inferior/posterior order. Each column corresponds to one dMRI measure. The associations with MAP are presented in the top row and PP in the bottom row. Coefficients for regions in the anterior circulation are presented as circles and those in the posterior circulation as triangles. All abbreviations are explained in the list of abbreviations in the main manuscript.

**Figure 2.**
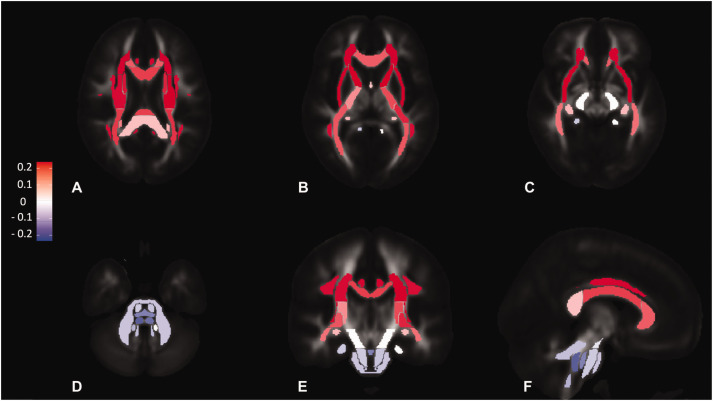
Standardised coefficients for concurrent MAP in fully adjusted linear models with MD as the outcome variable. a - axial slice at the level of the splenium of corpus callosum. b - axial slice at the level of the genu of corpus callosum, c - axial slice at the level of cerebral peduncle, d - axial slice at the level of the cerebellar peduncles, e - coronal slice at the level of the middle cerebellar peduncle, f - sagittal slice at the level of the superior cerebellar peduncle. Each region corresponds to one of the 27 tracts from the Juelich atlas. The colour scale encodes standardised coefficients.

In fully adjusted analyses, including the adjustment for PP, MAP remained strongly associated with white matter damage reflected by all diffusivity measures (Figure 1 and Supplementary Table 1). This effect was stronger in anterior circulation, but it was reversed in the posterior circulation and higher MAP was associated with reduced microstructural injury for all diffusivity measures. For example, the most positive associations between MAP and MD (Figure 2) were in the anterior and superior tracts of corona radiata and the most negative in the longitudinal brainstem tracts such as medial lemniscus and the superior cerebellar peduncle. The overall pattern was similar for MD, ICVF, and ISOVF, but the change of the directionality of effect was not demonstrated for FA and the effect on OD was larger in the posterior regions (top panel in Supplementary Figure 4, Supplementary Figure 5, Supplementary Figure 6, and Supplementary Figure 7).

MAP associations with increased white matter injury were independent of age (Supplementary Table 2). The effect of MAP on dMRI indices was stronger in older people (Supplementary Figure 8) due to a confounding effect of WMH load (Supplementary Figure 9 and Supplementary Table 2) as these interactions were attenuated in those in the lowest tertile of WMH (Supplementary Figure 10) and enhanced in those in the top tertile of WMH (Supplementary Figure 11). The regional interactions between age and the severity of WMH load were complex (Supplementary Figure 12 and Supplementary Figure 13). Consistent with predominance of WMH load within the anterior circulation, posterior regions were associated with greater microstructural injury in the absence of WMH (Supplementary Table 2).

### Interactions between diffusion markers and the pulsatile component of blood pressure

In unadjusted analyses, the effect size of PP on dMRI indices was larger than MAP. There were marked regional differences and the effect was stronger in the superior/anterior regions for MD, ICVF, and ISOVF and in the inferior/posterior regions for OD (Supplementary Figure 2). After adjusting for age and sex, the relationship between PP and dMRI indices in the anterior circulation greatly diminished or was no longer present due to the covariance between PP and age (Supplementary Figure 3).

**Figure 3.**
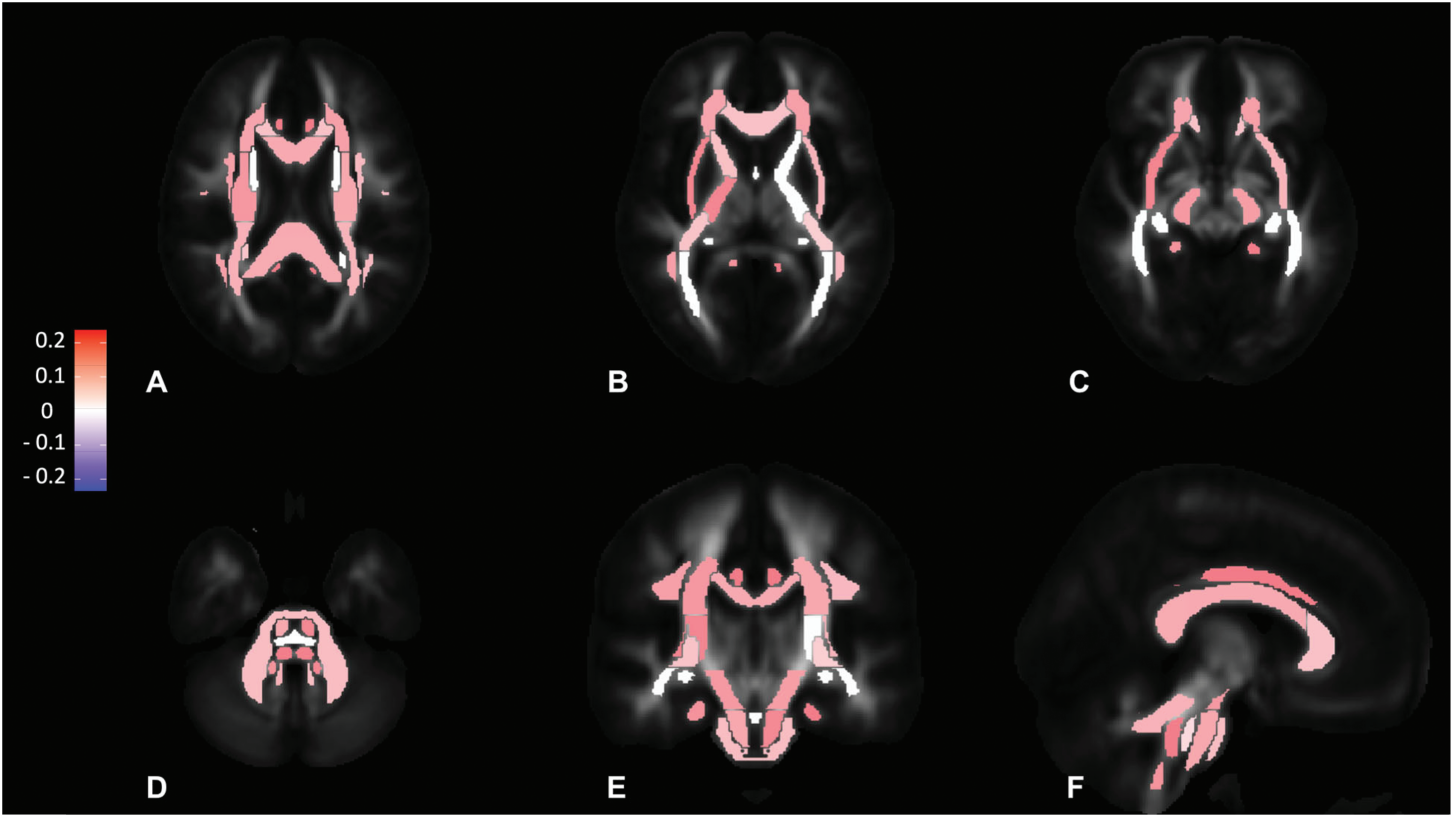
Standardised coefficients for concurrent PP in fully adjusted linear models with MD as the outcome variable. a - axial slice at the level of the splenium of corpus callosum. b - axial slice at the level of the genu of corpus callosum, c - axial slice at the level of cerebral peduncle, d - axial slice at the level of the cerebellar peduncles, e - coronal slice at the level of the middle cerebellar peduncle, f - sagittal slice at the level of the superior cerebellar peduncle. Each region corresponds to one of the 27 tracts from the Juelich atlas. The colour scale encodes standardised coefficients.

In fully-adjusted analyses, including an adjustment for MAP, PP was only weakly associated with white matter injury within the anterior circulation (Figure 1) for MD (Figure 3) and other dMRI indices (bottom panel in Supplementary Figure 4, Supplementary Figure 5, Supplementary Figure 6, and Supplementary Figure 7). Unlike MAP, associations between PP and dMRI indices of white matter damage in the posterior circulation were less attenuated in the fully adjusted analyses, and a reversal of the direction of the effect was only present for neurite density (ICVF) in some longitudinal tracts of the brainstem. Associations between PP and white matter injury were similar across age decades and WMH tertiles, with a slight reduction of the effect of PP on ISOVF with increasing age and WMH load (Supplementary Figure 8 and Supplementary Figure 9). Interactions between PP and age were significant only for MD and ICVF in those with high WMH load (Supplementary Figure 11).

## Discussion

The steady component of blood pressure represented by MAP and the pulsatile component represented by PP were both associated with microstructural white matter damage, independent of age and the extent of macrostructural white matter damage represented by WMH load. While MAP was associated with all analysed DTI and NODDI markers, both reflecting neurite density and organisation (FA, ICVF, OD) and overall diffusion and free water fraction (MD, ISOVF), PP was predominantly associated with the latter (MD and ISOVF). However, PP was most markedly associated with white matter injury in the posterior circulation for all indices after adjustment for MAP, except for higher neurite density in the brainstem’s long tracts, whereas MAP was generally protective against white matter injury in the posterior circulation regions, after adjustment for pulsatility and other confounders.

High blood pressure pulsatility is related to lacunar infarcts^20^ and higher WMH load, either directly or through the augmented effect of age,^21^ but the associations between haemodynamic factors within the anterior and posterior cerebrovascular circulation and white matter injury is well less established.^22^,^23^ White matter hyperintensities occur both in the supra- and infratentorial location,^24^ but at least in middle-aged people, white matter hyperintensities in the inferior/posterior regions are less common^25^ and diffusion imaging changes within the posterior circulation have not been extensively investigated. However, infratentorial cerebral microbleeds, but not lobar cerebral microbleeds, were associated with higher arterial stiffness^26^ whilst infratentorial aneurysms tend to have a higher rupture risk than anterior circulation aneurysms due to higher flow rates due to the lack of protective effect of the carotid siphon.^27^ This reflects anatomical and physiological differences between the anterior and posterior circulation, with likely less damping and more pulsatile blood flow due to longer and straighter cerebral vessels, which may be more vulnerable to stiffening with age;^11^ therefore, high PP may be more relevant to post circulation injury due to a greater risk of hypoperfusion and greater shear stress.

The association between MAP and white matter injury within the anterior circulation was stronger in older people and with an increase in WMH load, and WMH load confounded the interactions between dMRI with age. For example, the associations between MAP and reduced microstructural damage in the posterior circulation were present regardless of WMH load, and diminished with age, but the strong effect of MAP on MD and ICVF within the anterior circulation was attenuated in people in the lowest tertile of WMH load within their age decade. Age was strongly associated with all indices of white matter injury, to a greater extent than blood pressure, but whereas associations with MAP were independent of age and did not interact, PP interacted with age to enhance the association between age and MD or ICVF. Therefore, the observed changes may reflect processes observed in healthy ageing^6^,^28^,^29^ as well as pathology not present in healthy ageing,^9^ but the majority of regional effects remained after controlling for age and WMH load, which suggests that most of the dMRI changes represent pathological processes that are independent of age-related and macrostructural changes.^25,30^ Although antihypertensive use was associated with greater white matter injury for most indices, regardless of WMH load, there were no interactions with blood pressure, except for the interaction between PP and ICVF in the absence of WMH. This suggests that microstructural damage appears early in the course of hypertension and persists even if blood pressure is successfully controlled.^31^ dMRI measures are potentially useful as an early biomarker of SVD as MD in normal-appearing white matter (NAWM) of patients with SVD correlated with a decline in cognitive performance^32^ as well as physical function.^33^ The current analysis of the effect of blood pressure on the individual white matter tracts demonstrated that the negative effect of MAP on white matter integrity was most marked within the anterior circulation. MAP was associated with reduced tract anisotropy (FA) and neurite density (ICVF), indicating damage to the integrity of white matter tracts and with an increase in overall diffusivity (MD) and free water fraction (ISOVF) suggesting demyelination.^34^ In the posterior circulation, MAP was associated with reduced microstructural injury, represented by higher neurite density (ICVF) and lower MD, but the increase in FA and reduction in ISOVF were less consistent across the posterior white matter tracts. However, MAP seemed to be linked to higher neurite orientation dispersion (OD) within the posterior circulation. In contrast, PP was not associated with white matter injury markers of intracellular neurite density and orientation (FA, ICVF, OD), but only with markers dominated by extracellular free water (MD, ISOVF). The effect of PP was independent of WMH severity, but it enhanced the negative effect of age on MD and the extracellular compartment diffusion fraction (ISOVF). The regional differences in the effects of the steady and pulsatile component of blood pressure may explain why earlier studies suggested that dMRI changes reflect axonal loss and gliosis,^13^ axonal loss or dysfunction,^9^ reduction of white matter fibre organisation^35^ or an increase in free water due to oedema and demyelination without changes in the tissue compartment^34^ or due to plasma extravasation.^36^

The study has several strengths. Firstly, it is based on a large cohort of middle-aged community-based participants. Secondly, the study included not only normotensive and hypertensive people, but also those with mildly elevated blood pressure and with controlled hypertension, which are less often studied. Another advantage of this analysis is, that unlike earlier studies,^30^ it included both DTI and NODDI measures within individual white matter tracts, which made it possible to assess the interactions between MAP and PP and microstructural white matter integrity within specific anatomical regions and to compare the effect between the regions within the anterior and posterior cerebral circulation. Mixed effect modelling allowed us to investigate the association with blood pressure accounting for the differences between different types of tracts and for the different effect of WMH load.

It is also important to acknowledge the limitations of this study. Firstly, dMRI values included measures both from WMH lesions and NAWM. This might have potentially confounded the analysis and limited interpretation of the results, because, although high blood pressure is associated with dMRI changes in all white matter, they may be different in WMH and NAWM.^7^,^37^ However, analyses in patients in the bottom tertile of WMH load confirmed the main findings. Secondly, the automated method for WMH segmentation only results in estimates for cerebral white matter; therefore, an effect of infratentorial WMH on dMRI within the posterior circulation could not be investigated. As this study was based on reported mean dMRI values rather than diffusion images, there was no additional correction for susceptibility and pulsatile motion artefacts, which might have particularly affected inferior/posterior regions overlapping with the posterior circulation. Moreover, the cross-sectional nature of this study did not allow us to investigate the causal relationship between blood pressure and dMRI changes. In addition, the information regarding blood pressure lowering medication in the UK Biobank is based on self-reported data, with limited information available to investigate interactions between specific clasess of antihypertensives and cerebrovascular dynamics. Finally, the brachial pulse pressure used in this present study may not reflect intracranial arterial pulsatility.^38^

## Conclusions

This study demonstrated that both steady and pulsatile components of blood pressure were associated with microstructural white matter damage even in regions that are not common locations of WMH, with MAP in the posterior circulation having a protective effect against the negative effects of pulsatile blood pressure. These results showed that dMRI provides a way to study detailed microstructural changes and to better understand the mechanisms responsible for the development of white matter changes associated with raised blood pressure. These differences between the anterior and posterior circulations imply different haemodynamic mechanisms for chronic white matter injury and may therefore reflect differences in the associated regional risk of stroke and a need for personalisation of treatment strategies.
